# A Practical Treatise on the Diseases Peculiar to Women, Illustrated with Cases, Derived from Hospital and Private Practice

**Published:** 1845-04

**Authors:** 


*A Practical Treatise on the Diseases peculiar to Women, illustrated with Cases, derived from Hospital and Private Practice.* By Samuel Ashwell, M. D. Member of the Royal College of Physicians, London, and Obstetric Physician and Lecturer to Guy's Hospital. *First American from the last London edition. With notes,* by Paul B. Goddard, M. D., M. A. P. S., M. A. N. S., Lecturer on Anatomy, Philadelphia, &c., 8vo. pp. 520. Philadelphia, Lea & Blanchard, 1845.

